# COVID-19-related anxiety disorder in Iraq during the pandemic: an online cross-sectional study

**DOI:** 10.1186/s43045-020-00067-4

**Published:** 2020-10-26

**Authors:** Saad Kazim Karim, Perjan Hashim Taha, Nazar Mohammad Mohammad Amin, Habeeb Shuhaib Ahmed, Miami Kadhim Yousif, Ammar Mohmmed Hallumy

**Affiliations:** 1 Azadi Teaching Hospital, Nakhoshkhana Road 8, Duhok, Duhok Governorate, Kurdistan Region AM-1014 Iraq; 2 Stroke Unit, Wan Global Hospital, Duhok, Kurdistan Region Iraq; 3 College of Medicine, University of Duhok, Duhok, Kurdistan Region Iraq; 4 College of Medicine, University of Sulaimani, Duhok, Kurdistan Region Iraq; 5 General Surgery Department, Kufa Medical College, Kufa University, Kufa, Iraq; 6 Alzahraa College of Medicine, University of Basrah, Basrah, Iraq; 7 Medical City, Baghdad, Iraq

**Keywords:** Health-related anxiety, COVID-19, Anxiety, HAQ score, Fear of illness, Iraq

## Abstract

**Background:**

The COVID-19 outbreak is an unprecedented global public health burden, which popped up in China in late 2019 to early 2020 and distributed worldwide rapidly. Indeed, this pandemic transmission has raised global physical and mental health concerns. Mental health issues that concur with this public health emergency may pose anxiety, depression, and posttraumatic stress disorder. In Iraq, there are no registered known data on the psychological consequence of the public during the communicable disease outbreak. The ongoing study aims to address the paucity of these data as an appraisal of the mental health burden represented by anxiety disorder related to the global COVID-19 era.

**Results:**

Among the 1591 Iraqi respondents, 788 (49.5%) accounted for having health anxiety over the current home restriction situation. Younger ages experienced more COVID-19-related health anxiety compared to older ages. Females reported higher health anxiety compared to males (57.7% vs 42.3%). The health care professionals reported 20.9% health anxiety. The Iraqi southern population displayed more health anxiety compared to the northern and middle portions. This work showed about half of the respondents were spending over 60 min focusing on news of COVID-19. We found that 80 to 90% carrying out preventive efforts and home quarantine against COVID-19 infection. Interestingly, participants experienced fear from the risk of COVID-19 infection, whether more or equal to a level of war scare, in 70.1% of the sample.

**Conclusions:**

In Iraq, during the COVID-19 pandemic, nearly half of the respondents have health anxiety. Southern Iraqi cities displayed higher rates of anxiety. Also, being female, younger ages, holding an academic degree, or being a college student are associated with more prominent degrees of anxiety. Furtherly, it is important to adopt strategies for public health education and prevention and alerting future governmental responses focusing on psychological state impact among the general population.

## Background

The COVID-19 outbreak is an unprecedented global public health burden, which appeared in Mainland China in late 2019 to early 2020 and spread worldwide rapidly. The World Health Organization (WHO) recognized this outbreak as a Public Health Emergency of International Concern (PHEIC) that endangers international public health [1].

The unique property of the novel coronavirus, SARS-CoV-2, in its rapidity pandemic transmission has raised global physical and mental health concerns [2]. Moreover, mental health issues that coincide with this public health emergency triggered a wide variety of psychological problems, such as panic disorder, anxiety, depression, posttraumatic stress disorder, and an increased self-blame [3]. In comparison, COVID-19 imposes more asymptomatic transmissible contagious disease than SARS which may lead to diminished mental well-being and exacerbates the increased risk of negative feelings [4].

It is noteworthy that rapid overpowering news, followed by great psychological pressure not only in COVID-19-confirmed cases but extended to suspected individuals [5]. Moreover, worry toward health is one of the challenges among general health workers and frontline health professionals. Health care workers are not only afraid of getting infected but also worried about contaminating their families, friends, and colleagues with the virus [6]. Further, the collapse of industries, financial losses, and unemployment risk are other provocations that could enhance the negative emotion of the affected individuals [7].

Health anxiety denotes to a continuum of behaviors and cognitions of an individual’s response that encompasses persistent worries about an illness that extend from mild to severe forms and can belong to the hypochondriasis, based on DSM-IV [8]. Multiple risk factors reported being of considerable influence on health anxiety evolution include personality and mood characteristics [9]. Similarly, the potential for adaptation is through the ability to withstand emotional and physical distress.

In Iraq, there was little or no extant registered data on the psychological consequence and health anxiety per se in the public during the peak of communicable disease outbreak such as H1N1 in 2009. Thus, the current study aims to address the paucity of these data as an estimation of the mental health burden during this universal outbreak of COVID-19.

Since the government took rigorous lockdown measures to contain the epidemic, positive trends have been seen. Total curfew started from March 17, 2020, to the partial lifting on April 26 with keeping airport closures extended. On February 24, the first coronavirus case was reported, and the first death was on March 4, 2020. However, the total was 1761 confirmed cases and 1224 recovered by April 26, 2020, extended [10]. Therefore, in this exceptional circumstance, the researchers adopted the aim of recognition of health anxiety among a sample of the Iraqi population during the COVID-19 pandemic.

The goal of this study is to survey the general community in Iraq to identify the prevalence rate of possible health anxiety among the gathered sample during the COVID-19 pandemic, measure the differences of health anxiety rates and its factors among the people of different parts of Iraq, evaluate the responses to specific items on fear of COVID-19 virus infection, and estimate the correlation between COVID-19-specific anxiety and Health Anxiety Questionnaire (HAQ) scale and its subscales.

## Method

### Study design and participants

We emphasized a cross-sectional online survey via English-Arabic combined type of questionnaire formulated by the researchers and uploaded via the web-based Survey Monkey® platform [11]. The inquiries were released randomly through announcement links on social media platforms to reach participants distributed all over the19 Iraqi provinces. The self-reported structured questionnaire consisted of questions that covered three groups of inquiries: (1) health anxiety assessment, (2) knowledge and concerns about COVID-19, and (3) sociodemographic data. It was composed of 42 multiple choice and short answer questions. Data collection took place over 23 days (March 21 to April 12, 2020) from our convenient sample which constituted 1591 engaged respondents, and it took on average 8 min to reply to all questions. Ethical approval was obtained from the Scientific Committee at the College of Medicine, University of Duhok.

### Scales and assessment

For the identification of worry and fear toward the individual’s health, we used the HAQ. It is quick, reliable, self-administered, sensitive to nonoccurrence of events, and validated in community samples, with a simple modification to fulfill the COVID-19 situation [12]. The questionnaire consists of 21 questions with 4-point rated scales [13]. The scale is composed of 4 factor subscales: health worry and preoccupation, fear of illness and death, reassurance-seeking behavior, and interference with life [14]. The cutoff score of 20 was used to determine possible health anxiety. Additionally, we adopted COVID-19 anxiety-specific responses by three questions concerned with (1) the time spent focusing on COVID-19 in a day, (2) how much COVID-19 scares compared to fear from war, and (3) agreeing on the treatment of COVID-19 anxiety.

### Statistical analysis

Descriptive statistics were used to depict the sample’s sociodemographic characteristics. Frequencies and percentages of responses were displaced for each variable for 1591 responses. Iraq governorates were divided geographically into 3 groups: (a) the northern governorates, (b) the middle governorates, and (c) the southern governorates. The one-way ANOVA test was employed to compare the means and the bivariate correlations to measure the correlations between different scales and subscales of the instrument used. The internal consistency for testing reliability of HAQ by Cronbach’s alpha coefficient was checked (*α* = 0.876) which is high. The significance level of *P* value < 0.05 is significant; *P* value < 0.001 is highly significant. The analysis of data was performed using SPSS Statistic 22.0 [15]. Besides, this study complies with the ethical rules of the 64th World Medical Association Declaration, General Assembly, Helsinki (2013) [16].

## Results

Table 1 shows the descriptive statistics of the sample. Younger ages and singles were more prevalent in our sample. Genders were approximately equally distributed. About half of the participants had some or completed academic degrees. The unemployed participants constituted 4.6% of the whole group. Regarding residence, the urban inhabitants were the most common constituting 87.2% of the total. About half of the sample were living in households comprising 3–5 persons. Further, we noticed that 7.8% of the respondents displayed a history of chronic respiratory diseases.

**Table 1:** Sociodemographic data of respondents (*N* = 1591)

Item	Number	Percent
**Age**		
18–24 years	613	38.5
25–34 years	376	23.6
35–44 years	331	20.8
45–54 years	166	10.4
55–64 years	76	4.8
65+ years	29	1.8
**Gender**		
Male	767	48.2
Female	823	51.7
**Marital status**		
Single	644	40.5
Married	281	17.7
Divorced or separated	15	0.9
Widowed	12	0.8
**Educational attainment**		
None	16	1
Primary school	16	1
Lower secondary school	26	1.6
Higher secondary school	207	13
University degree or some	833	52.4
Master’s degree	171	10.7
Doctorate or equivalent	312	19.6
**Occupation**		
Employed	190	11.9
Unemployed	73	4.6
Health care professional	402	25.3
Student	599	37.6
Teacher	218	13.7
Others	99	6.2
**Residence**		
Urban	1387	87.2
Suburban	158	9.9
Rural	46	2.9
**Household size**		
1 person	21	1.3
2 persons	85	5.3
3–5 persons	789	49.6
6+ persons	695	43.7
**History of chronic respiratory disease**		
No	1467	92.2
Yes	124	7.8
**Total**	1591	100

The frequencies and percentages of possible health anxiety are presented in Table 2. Among the 1591 respondents to the online survey, 788 (49.5%) expressed health anxiety. Health anxiety is more common in younger ages comparing to old ages (43.7% vs 1.5%). Females reported more health anxiety (57.7%) in contrast to males (42.3%), and the findings are significant (*χ*^2^ = 22.375, df = 1, *P* < 0.001). University students or graduates showed a higher percentage of health anxiety (56.5%) comparing to other educational attainment groups (*P* < 0.001). Those holding university degrees or are university students recorded higher percentages of health anxiety. Health anxiety rates were more prevalent in health care professionals with a *P* < 0.001 and low in patients having chronic respiratory illnesses (*P* < 0.001). Marital status, residence, and household size do not show any significant differences regarding rates of health anxiety between their categories.

**Table 2:** Frequencies and percentages of possible health anxiety among Iraqi participants (*N* = 1591)

Demographic variable	No. of cases	% of cases	***χ***^**2**^	df	***P*** value
**Age**			33.764	5	< 0.001**
18–24 years	344	43.7			
25–34 years	198	25.1			
35–44 years	146	18.5			
45–54 years	62	7.9			
55–64 years	26	3.3			
65+ years	12	1.5			
**Gender**			22.375	1	< 0.001**
Male	333	42.3			
Female	455	57.7			
**Marital status**			6.171	3	0.104
Single	354	68.3			
Married	144	27.8			
Divorced or separated	10	1.9			
Widowed	10	1.9			
**Educational attainment**			39.130	6	< 0.001**
None	7	0.9			
Primary school	11	1.4			
Lower secondary school	18	2.3			
Higher secondary school	113	14.3			
University degree or some	445	56.5			
Master’s degree	78	9.9			
Doctorate or equivalent	111	14.1			
**Occupation**			29.834	5	< 0.001**
Employed	104	13.3			
Unemployed	38	4.9			
Health care professional	164	20.9			
Student	337	43			
Teacher	100	12.8			
Others	40	5.1			
**Residence**			294	2	0.863
Urban	688	87.3			
Suburban	79	10			
Rural	21	2.7			
**Household size**			5.306	3	0.257
1 person	6	0.8			
2 persons	39	4.9			
3–5 persons	396	50.3			
6+ persons	347	44			
**History of chronic respiratory disease**			8.498	1	0.004*
No	711	90.2			
Yes	77	9.8			
**Total health anxiety diagnosis**					
No	803	50.5			
Yes	788	49.5			

*χ*^*2*^ chi-square test, *df* degree of freedom

*P < 0.05 is significant; **P < 0.001 is highly significant

**Fig. 1:**
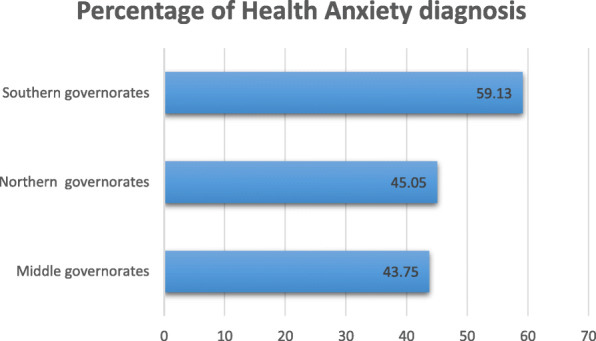
Percentages of possible health anxiety (by HAQ) concerning COVID-19 among participants of governorates of 3 geographical parts of Iraq (*N* = 1591)

Figure 1 displays the percentages of possible anxiety measured by HAQ concerning COVID-19 among the samples of three geographical parts of Iraq: northern, middle, and southern. The possible health anxiety diagnosis was more prevalent in the southern governorates (59.1%) compared to the northern and middle parts of Iraq (45.1% and 43.8%, respectively).

Table 3 demonstrates the means and standard deviations of the measures applied in our study between the participants of governorates of three geographical parts of Iraq. The sample group of southern governorates presented with higher means of health anxiety and its four subscale means compared to the northern and middle sample groups. The differences were either significant or highly significant.

**Table 3:** Means and standard deviations of measures between the participants of governorates of 3 geographical parts of Iraq (*N* = 1591)

Scales and subscales	Northern governorates, M (SD)	Middle governorates, M (SD)	Southern governorates, M (SD)	***F***	df	***P*** value
Total HAQ	0.89 (0.44)	0.9 (0.47)	1.07 (0.49)	25.459	2	< 0.001**
Health worry and preoccupation	0.92 (0.51)	0.94 (0.54)	1.09 (0.56)	17.820	2	< 0.001**
Fear of illness and death	0.94 (0.57)	0.94 (0.58)	1.14 (0.62)	20.341	2	< 0.001**
Reassurance-seeking behavior	1.08 (0.65)	1.1 (0.64)	1.22 (0.67)	7.379	2	0.001*
Interference with life	0.52 (0.62)	0.51 (0.57)	0.71 (0.75)	15.360	2	< 0.001**
Specific COVID-19 anxiety	1.69 (0.47)	1.57 (0.51)	1.68 (0.45)	6.636	2	0.001*

*M* mean, *SD* standard deviation, *F* one-way ANOVA, *df* degree of freedom

*P value < 0.05 is significant; **P value < 0.001 is highly significant

Table 4 shows the responses to our questionnaire’s specific items about fear of COVID-19. Approximately half of the participants spend more than one hour per day focusing on COVID-19. Most of the participants constituting 93.7% and 82.3% are practicing preventive measures against COVID-19 and home quarantine, respectively. The results on this table show that 70.1% were scared from COVID-19 to the degree equal to or even higher than being scared from a war.

**Table 4:** Responses to specific items on fear of COVID-19 virus infection among participants (*N* = 1591)

Question	Number	Percent
Time spent focusing on COVID-19 (more than 1 h/day)	769	48.4
Practicing preventive measures against COVID-19 infection	1490	93.7
Practicing home quarantine against COVID-19 infection (often or mostly)	1310	82.3
How much COVID-19 scares (more or equal to the level of war scare)	1115	70.1
Agreeing on the need to treat COVID-19 anxiety	446	28

Table 5 displays the correlations between specific COVID-19 anxiety mean and total health anxiety scales and subscales. The specific COVID-19 anxiety questions are covered by responding to three specific questions: (1) how much COVID-19 scares compared to fear from war, (2) the time spent focusing on COVID-19 in a day, and (3) agreeing on the treatment of COVID-19 anxiety. The findings demonstrated that specific COVID-19 anxiety item means are positively correlated with the total health anxiety scales and its four subscales. The degree of correlation with total HAQ score and with its two subscales (health worry and preoccupation, and fear of illness and death) is moderate and is weakly positive with the remaining two subscales (reassurance-seeking behavior and interference with life). The total HAQ scale with its 4 subscales in addition to the subscale correlations between each other displays positive correlations of different strengths shown in this table. Interestingly, the total HAQ score is perfectly correlated with the health worry and preoccupation subscale (*r* = 0.909, *P* < 0.001), and the correlation is strong with the remaining three subscales (*r* lies between ± 0.50 and ± 1, *P* < 0.001). Additionally, there is a very strong positive correlation between the health worry and preoccupation subscale and the fear of illness and death subscale (*r* = 0.711, *P* < 0.001). All other subscales are positively correlated in various degrees.

**Table 5:** Correlations between COVID-19-specific anxiety and HAQ scale and subscale scores (*N* = 1591)

	1	2	3	4	5	6
COVID-19-specific questions	1					
Health worry and preoccupation	0.451**	1				
Fear of illness and death	0.391**	0.711**	1			
Reassurance-seeking behavior	0.292**	0.496**	0.421**	1		
Interference with life	0.251**	0.409**	0.333**	0.263**	1	
Total HAQ score	0.467**	0.909**	0.876**	0.641**	0.567**	1

**P value < 0.001 is highly significant

## Discussion

The 2019 novel coronavirus (COVID-19) has made to a quick and serious outbreak of threatening respiratory disease, which originated first in China and has expanded as a worldwide pandemic, with far-reaching effects [17]. The emergence of this virus is regarded as a particular stressor for people’s physiological, psychological, and behavioral reactions [18]. Moreover, anxiety is one of the self-regulation subjective capabilities as a response to COVID-19 that will have a psychological impact on the well-being of the general population [19]. The limited knowledge of the COVID-19 and the upsetting news may lead to anxiety and fear in society [5]. It is notable that our study is the first one holding in Iraq to examine the health anxiety related to COVID-19 by an online survey method of data collection.

In our study, among the 1591 respondents to the online survey, 788 (49.5%) accounted for having health anxiety. The former history of the Iraqi population going through decades of internal and external conflicts making it easy to believe that mental disorders are common among the Iraqi population. In turn, this is forcing them to be more vulnerable to anxiety disorders and may explain the higher prevalence encountered in our study [20]. Another reasonable explanation may be that the Iraqi people believe that inadequate public health care services and weak health systems being unable to manage the probable upcoming pandemics. Moreover, the current world-wide home restriction situation, including the measures taken in Iraq, obligated people to be more exposed to an unheard-of stressful position of unknown duration [21]. Iraqi measures included (a) awareness of the seriousness of the situation started at the beginning of March 2020 and (b) prevention and control measures of movement suspension were announced in some cities of Iraq that have positive COVID-19 cases. Following the mid-term of March 2020, more restrictions imposed on traffics and strong isolation measures began after March 20, 2020, especially after the detection of further COVID-19 cases.

Many other studies proved the psychosocial reactions of the general population toward the severe acute respiratory syndrome outbreak [22]. The mutual topics in these psychological reactions included anxiety, depression, posttraumatic stress, and stigmatization. Comparing to other studies, an online survey on the psychological impact of COVID-19 in China estimated the rate of anxiety being 36.4% with different severities, including 28.8% having moderate to severe anxiety [23].

Our survey identified that younger ages experienced more COVID-19-related health anxiety compared to older ages. Analogous data were proven by a study in China in which the anxiety risk of people above 40 years old was 0.40 odds ratio (CI 0.16–0.99) times compared with those below 40 years old [24].

In this work, females reported higher health anxiety compared to males (57.7% vs 42.3%). An analysis in China on anxiety and depression rates among the general population revealed that females’ anxiety risk was 3.01 times equated to males [24]. Similarly, the anxiety scores of female medical staff also were higher compared to males [25]. Studies proved that the fear and rousing responses are more active among females, and this can explain the observed gender differences [26].

Respondent Iraqi students expressed a notifiable fraction (43%) of possible anxiety, especially college students, during the span of extending outbreak. To some degree, parallel results were shown in a survey from Changzhi Medical College, which indicated that 24.9% of the students were impaired with received anxiety over the COVID-19 outbreak era [27]. Of these students, 0.9% experienced severe anxiety, and 21.3% experienced mild anxiety. Accordingly, college students who experienced anxiety undoubtedly will have a negative impact on the educational process and possible delays in education. Likewise, our study showed that health care professionals have a higher percentage of health anxiety next to the student group. This is consistent with previous studies which showed that health professionals are suffering from mental health problems during outbreaks.

The health care professionals reported 20.9% health anxiety, which seems to be near some other studies’ rates. A previous survey showed that the incidence of anxiety among medical staff was 23.04% during the COVID-19 epidemic [25]. In contrast, some other studies evidenced much higher percentages of anxiety reaching up to 44.7% [5]. Reasonable elucidation is that they have the responsibility of attention to the patients, obligatory handling of patients, and to a lesser extent contact with patients’ families or relatives [5]. Additionally, frontline health workers usually have close contact with infected patients, excessive workload, and isolation, making them highly vulnerable to experience physical exhaustion, worry, mood problems, and sleep disturbances. The proportionally low level of our figure compared to the last-mentioned study could be attributed to the low toll of confirmed COVID-19 cases at the time of initiation of the survey, which was less than 300 patients.

Although our findings were not statistically significant regarding anxiety linkage with household size, the more households were accompanying to more health anxiety percentages. However, as much as the family size or the household size increase, the thoughts of the danger of exposure to COVID-19 are rising not only for themselves alone but extends to their colleagues and their families. Furthermore, people must beguile to the demands of elderly parents who are in the huge need to support, in addition to the needs of children who are abruptly out of school and exposed to movement bans [28].

Unexpectedly, the rate of health anxiety in those having a history of chronic respiratory disease was low. The smaller sample size and lower proportion of those having chronic respiratory illnesses might affect the results. However, at the time of the sample collection, there was an uncertainty of population knowledge about the definite magnitude of the risks of unfavorable outcomes attributable to COVID-19 in patients with chronic respiratory diseases.

In this survey, based on the regional distribution of our sample, the southern Iraqi population displayed more health anxiety compared to the northern and middle portions. The respondent from southern provinces demonstrated significantly higher health-related anxiety (59.1%) than the middle or northern provinces. Furthermore, the difference in distribution between these regions was not only in the means of diagnosis of health anxiety but also broaden to the four defining factors or subclasses outlined as health worry and preoccupation, fear of illness and death, reassurance-seeking behavior, and interference with life. Unlikely, an equivalent survey done in China deduced that there are no differences between the midwestern and eastern regions [24]. This might be explained by that the southern cities exhibit more social conflicts and border insecurity, which may subsequently submit a significant awareness attitude about this accused outbreak. It is worthy to mention that the rise of infection-related reaction has been a common response when people are threatened with an infection that originates from outside of their community [18].

The current study poses an impression of how COVID-19-related anxiety affects the Iraqi general population as they practiced a specific comprehensive precautionary measure against COVID-19 infection. Among them, about half of the respondents were spending more than 60 min focusing on news of COVID-19. This impact has got to be compatible with the results from a Chinese web-based cross-sectional study, in which 36.3% of participants consumed 1–2 h to follow COVID-19 news [29].

Also, this survey expressed that 80 to 90% carrying out preventive measures and home quarantine against COVID-19 infection which is similar to other studies performed during the COVID-19 outbreak in China [30]. Hence, this success in self-practiced prevention hypothetically may concern about changing the epidemiological curve of COVID-19 cases in Iraq compared with that in China, Italy, and the UK [31]. This perceived threat could act as a motivational influence to perform a behavior that facilitates COVID-19 prevention [32].

Iraq has high prevalence rates of mental health crises as a result of previous wars, violence, and oppression [33]. Noticeably, the data from this work disclosed that the participants’ experienced fear from the risk of COVID-19 infection was either more or equal to the level of war scare, in 70.1% of the sample, while data from Germany demonstrated that 62% of answered people had general worry about COVID-19 [34].

As aforementioned, it is argued to put a specific scale of fear from COVID-19 [32]. Our study evidenced that specific COVID-19 anxiety item means are positively correlated with the total health anxiety scales and its four subscales. Additionally, these positive correlations and high anxiety levels are manifested between the used subscales.

### Limitations

Illness-related anxiety was not among the exclusion criteria; therefore, cases diagnosed as having health anxiety might include patients who previously had illness-related anxiety. However, previous surveys on the Iraqi population demonstrated only 13.8% to have anxiety disorders [20]. Response bias may be considered as one of these limitations since the response rate from the capital Baghdad was only 9% which may be attributed to either too stressed to respond or not interested in this survey. The sample was convenient, and the online participation has a possibility of selection bias. The response of the people took longer than expected which could interfere with the study being cross-sectional which was due to the fact that the authors did not have access to people from all over Iraq, to begin with. This problem was later solved by involving co-authors from the governorates that had low responses.

## Conclusion

In Iraq, during the COVID-19 pandemic, nearly half of the respondents rated to have health anxiety. Southern Iraqi cities displayed higher rates of anxiety. Among the Iraqi population, being female, younger ages, academic professionals, university students, and health care workers associated with more prominent degrees of anxiety. Its appraisal is to adopt proper strategies for public’ health education and crucial focusing on coping strategy through the general population.

## Data Availability

The datasets that were generated during and/or analyzed during the current study are available from the corresponding author on reasonable request.

## Abbreviations

COVID-19: Coronavirus disease 2019
SARS: Severe acute respiratory syndrome
DSM-IV: Diagnostic and statistical manual of mental disorders, fourth edition
H1N1: Influenza A (H1N1) virus or swine flu
HAQ: Health Anxiety Questionnaire
